# Validation of the Eating Habits Questionnaire in Greek adults

**DOI:** 10.14806/ej.28.0.1029

**Published:** 2023-05-16

**Authors:** Georgia Bali, Ioulia Kokka, Fragiskos Gonidakis, Eleni Papakonstantinou, Dimitrios Vlachakis, George P. Chrousos, Christina Kanaka-Gantenbein, Flora Bacopoulou

**Affiliations:** 1 Postgraduate Course of Stress Science and Health Promotion, School of Medicine, National and Kapodistrian University of Athens, Athens, Greece; 2 Eating Disorders Unit, First Department of Psychiatry, School of Medicine, National and Kapodistrian University of Athens, Athens, Greece; 3 Genetics Laboratory, Biotechnology Department, School of Applied Biology and Biotechnology, Agricultural University of Athens, Greece; 4 Center for Adolescent Medicine and UNESCO Chair on Adolescent Health Care, First Department of Pediatrics, School of Medicine, National and Kapodistrian University of Athens, Aghia Sophia Children’s Hospital, Athens, Greece

## Abstract

Healthy eating has gained ground in people’s daily lives in modern society. However, an overwhelming preoccupation with healthy eating can lead to a pathological form setting the ground for orthorexia nervosa. This study aimed to validate the Greek version of the Eating Habits Questionnaire (EHQ) in adults 18 to 65 years old. The EHQ evaluates orthorexia nervosa traits. An online survey was conducted among adults of the general Greek population by administrating a battery of self-report instruments. The IPIP Big-Five personality questionnaire, Beck’s Depression Inventory, the Obsessive-Compulsive Inventory-Revised, the Bulimic Investigatory Test, the Edinburg BITE, and the Eating Attitudes Test-13 were used. Internal consistency, test-retest reliability, and convergent and criterion validity were examined. A total of 551 adults (92.2% females) voluntarily participated in the study. Results suggest that the Greek version of the instrument has good psychometric properties. Analysis revealed a 3-factor model explaining 48.20% of the total variance. Cronbach’s alphas ranged between 0.80 to 0.82, indicating good internal consistency. The test-retest reliability analysis showed no statistically significant difference between the measurements of the first and the post-2 weeks. Correlations with other eating disorder-related constructs were found to be weak to moderate. Body mass index was not significantly correlated with neither of the three EHQ subscales. The Greek version of EHQ is a robust instrument that could be used in clinical practice and research in the field of eating disorders in Greece.

## Introduction

The preventive and therapeutic role of healthy eating in numerous diseases of recent times has resulted in an on-growing interest of both scientists and patients (Kiss-Leizer and Rigó, 2019). Despite the positive outcomes of this otherwise beneficial behavior, in certain cases it might be considered as a social trend that leads to extremities (Douma *et al*., 2021). In other words, when healthy eating becomes obsessive, psychological disturbances may emerge. This overwhelming preoccupation can become the main life-priority, and set the ground for orthorexia nervosa (ON). Borrowed from the Greek language, the prefix -ortho means “correct” (Gleaves *et al*., 2013). An individual with ON attempts to reach optimum health through strict dietary practices. The central axis is not the quantity of food, but its perceived quality. Among the behavioral patterns exhibited by those with ON, the consumption of solely what is perceived as healthy and “pure” food, the inflexible dietary rules, and specific for each individual rituals during food processing and meal preparation are included. The obsessive nature of ON can lead individuals to pursue even more rigid diets such as veganism, raw foodyism, and fruitarianism (Hayes *et al*., 2017). Consequently, this fixation on healthy eating results in clinically significant impairments, such as social withdrawal, nutrient deficits, or even severe malnutrition, and subsequently in impaired quality of life. ON has been found to relate with attachment issues (Barnes and Caltabiano, 2017), perfectionism (Brytek-Matera *et al*., 2020), narcissism (Oberle *et al*., 2017), obsessive-compulsive features (McComb and Mills, 2019) and excessive exercise (Rudolph, 2018; Oberle *et al*., 2018), and less commonly with depressive symptomatology, sex (Strahler, 2019), and body mass index (BMI) (Brytek-Matera *et al*., 2020).

ON is not included in mental health classification manuals (American Psychiatric Association, 2013). Currently, it falls into the group of Eating Disorders Not Otherwise Specified (EDNOS), as it appears to have similarities with avoidant/restrictive food intake disorder (ARFID), anorexia nervosa (AN) and obsessive-compulsive disorder (OCD) (American Psychiatric Association, 2013). Although standardized clinical diagnostic criteria are not established, researchers of the field identify the pathological fixation with healthy eating, emotional consequences due to self-imposed rules, and functional impairment across various areas of life as the main criteria (Cena *et al*., 2019). The ‘Eating Habits Questionnaire’ (EHQ) was developed by David H. Gleaves and colleagues to assess symptoms of orthorexia nervosa. It is a 21-item orthorexia test comprised of three subscales evaluating the individual’s knowledge on healthy eating, and detecting emotional attitudes and other problems towards healthy eating, exhibiting good psychometric properties (Gleaves *et al*., 2013). The present study aimed to translate and validate the Greek version of EHQ in the general population, to obtain a tool for the assessment of adult orthorexia characteristics.

## Materials, Methodologies and Techniques

### Translation procedure

The translation procedure was initiated after receiving permission by the first author of the EHQ, and followed several steps, as indicated by similar research (Kokka *et al*., 2021). The questionnaire was translated in Greek by a mental health professional specialized in eating disorders. The translation aimed more at a conceptual rather than a linguistic equivalent. The Greek version of EHQ questionnaire was back-translated by an independent professional translator with no knowledge of the original questionnaire or the purpose of the study. The back-translated version was evaluated by a bilingual speaker to identify any possible inconsistencies and proceed with the final version of the questionnaire. Following, a pre-test it was administrated to a sample of 10 volunteers to examine the clarity of each question. No changes were required and the final Greek version of the EHQ questionnaire was obtained.

### Participants and study design

The survey was conducted online via several social media platforms. Researchers aimed to at least 10 responses per item as suggested by previous validation efforts (Costello and Osborne, 2005). A response was considered eligible for analysis if the respondent was 18 to 65 years old and a Greek speaker. Responses with missing values were excluded from analysis. No additional inclusion or exclusion criteria were applied. To examine the test–retest reliability of each scale, 2 weeks later, 31 of the participants completed the 21-item EHQ to assess the repeatability of the scale across time.

### Ethical considerations

The study followed the ethical standards of the Declaration of Helsinki. The study protocol was approved by the scientific committee of the “Science of Stress and Health Promotion” Master’s Program of the School of Medicine, National and Kapodistrian University of Athens, Greece (45274-31/8/2020). Eligible participants were extensively informed about the study’s purpose with a description of the research protocol before completing the questionnaires. Submission of the response was considered an automatic consent. Participation in the study was not compensated. The volunteers participating in the retest reliability procedure were initially informed in detail and agreed to register and provide their emails for further communication.

### Measures

Data were collected using a battery of self-report questionnaires and a demographics’ form.

Demographics questionnaire: this included sex, age, educational level, marital status, and history of an eating disorder. Data of current weight and height were requested to calculate the body mass index (BMI).

Self-report on weight satisfaction: participants were asked to respond to the degree to which they were satisfied with their current weight on a 3-point scale ranging from ‘Not at all’ to ‘Absolutely’. Weight satisfaction was examined by the question “Are you satisfied with your current body weight?”, and responses were given on the same 3-point scale.

Eating Habits Questionnaire (EHQ): EHQ is used for the evaluation of orthorexia nervosa traits. It consists of 21 items answered on a 4-point Likert-type scale (1: False, not at all true, 4: Very true) and examines three different factors; knowledge of healthy eating (5 items), problems associated with healthy eating (12 items), and feeling positively about healthy eating (4 items) (Gleaves *et al*., 2013).

IPIP Big-Five personality questionnaire: this instrument is used for the identification of personality traits. It consists of 50 items and responses are given on a 5-point Likert-type scale (1=Very Inaccurate to 5=Very Accurate) and examines the five aspects of the personality; extraversion (10 items), agreeableness (10 items), conscientiousness (10 items), emotional stability (10 items), and intellect/ imagination (10 items). The Greek version of IPIP exhibits good construct validity, internal consistency, and concurrent validity (Ypofanti *et al*., 2015).

Beck’s Depression Inventory (BDI): BDI is used for the evaluation of psychological and physical symptoms of depression. It consists of 21 items and total score ranges from 0 to 13 indicating minimal depression, 14 to 19 indicating mild depression, 20 to 28 indicating moderate depression and 29 to 63 indicating severe depression (Beck *et al*., 1988). The Greek version of BDI exhibits good psychometric properties (Giannakou *et al*., 2013).

Obsessive-Compulsive Inventory-Revised (OCI-R): OCI-R is used for the evaluation of OCD symptomatology. It consists of 18 items and examines the six subtypes of obsessive spectrum: washing, checking, ordering, hoarding, obsessing and neutralizing. Responses are given on a 5-point Likert-type scale (0=Not at all to 4=Extremely). Scores higher than 30 indicate characteristics of the obsessive-compulsive disorder (Foa *et al*., 2002). The Greek adaptation exhibits excellent internal consistency and good convergent, and divergent validity (Simos *et al*., 2019).

Bulimic Investigatory Test, Edinburg (BITE): BITE is applied for the evaluation of bulimic symptomatology severity. It consists of 33 items and two subscales; symptoms, and symptom severity. The cut-off score for the symptom subscale is 15, while for the severity of symptom subscale 5. The instrument has been translated in the Greek language for research purposes (Henderson and Freeman, 1987).

Eating Attitudes Test-13 (EAT-13): EAT-26 is used for the evaluation of maladaptive behaviours and attitudes associated with anorexia nervosa (Garner *et al*., 1982). The adaptation of the EAT-26 in the Greek population revealed a new 13 item EAT model, in which responses are given on a 6-point Likert-type scale (1=Always to 6=Never). It examines three different factors; dieting (4 items), food preoccupation (6 items), and important others (3 items) (Douka *et al*., 2009).

### Data analyses

Descriptive analyses were used to calculate frequencies (%) for categorical variables and median (IQR) and mean (SD) for continuous variables. Exploratory Factor Analysis (EFA) on item-level was implemented to assess the three-factor structure of EHQ. The Kaiser-Meyer-Olkin Measure of Sampling Adequacy (KMO) and Bartlett’s Test of Sphericity were utilized to examine sample’s adequacy and the adequacy of the correlation between items, respectively. Principal component analysis (PCA) with rotated factor loadings was performed to evaluate the internal structure of the measure. Eigenvalues greater than 1 determined the number of subscales. Each factor included only items with loadings greater than 0.3. Cronbach’s alpha values were extracted to estimate internal consistency for the three subscales. The Spearman’s rho correlation coefficient was used to examine the inter-correlation between the three subscales. Total scores and subscale scores of instruments were calculated and correlated with the EHQ subscales scores. Association between EHQ subscales and other study measurements were performed by Mann-Whitney, Kruskal Wallis and One way ANOVA tests. The Wilcoxon signed-rank test was used to examine the test-retest reliability of the scale across time. Statistical analysis was performed using the Statistical Package for Social Sciences (SPSS) for Windows (version 25) statistical software (SPSS Inc., Chicago, IL).

## Results

The basic demographic characteristics of the participants, as well as the results for all the questionnaires and their subscales are presented in Table 1. A total of 551 adults from the general population participated in the survey, while 508 (92.2%) were females. Mean scores of the questionnaires indicated that the sample demonstrated high levels of OCD symptomatology (>21), tendency to exhibit an eating disorder (>12), possible presence of bulimia, but no disturbing depression levels (<16).

### Exploratory factor analysis (EFA)

The rotated factor loadings of the principal components analysis (PCA) with direct oblimin rotation are presented in Table 2. The value of the Kaiser-Meyer-Olkin (KMO) coefficient was 0.880 which verified that the sample was adequate. Bartlett’s Sphericity Test (χ2) was 4056.685, p<0.0001, which indicated that correlations between items were sufficiently large to perform PCA. All three subscales had eigenvalues greater than the 1 and in combination explained 48.20% of the variance. All items had factor loadings >0.3.

Cronbach’s alpha values were calculated to explore the internal consistency of the EHQ subscales. Results demonstrated good internal consistency with coefficient alpha values ranging from 0.80 to 0.82. Alpha coefficient was 0.80 for ‘Knowledge’ subscale, 0.82 for ‘Problems’ subscale, and 0.82 for ‘Feelings’ subscale. Correlations between the EHQ subscales were moderate and ranged from 0.44 to 0.52 indicating that elements of the subscales were positively correlated to each other, measuring the same construct without excess (Table 3).

### Test-Retest Reliability

The descriptive characteristics of the EHQ and the three subscales, from both first and second measurement points are presented in Table 4. Out of 551 respondents who completed the initial test, 21 subjects agreed to a post-2 weeks second testing to examine the test-retest reliability. The Wilcoxon signed-rank test was used to examine the test-retest reliability of the 3 subscales. No statistically significant difference was found between the two measurement points (p>0.05 for each subscale).

### Convergent and Criterion validity

The associations between EHQ subscales and other study variables are presented in Table 5. Educational level was significantly associated with the EHQ ‘Knowledge’ subscale, while marital status with the ‘Problems’ subscale (p=0.016 and p=0.015 respectively). To evaluate criterion validity, the EHQ was examined in relation to other instruments measuring related constructs; personality traits, depressive symptomatology, OCD symptoms, maladaptive eating behaviours and bulimia traits. From IPIP evaluation, agreeableness and intellect/imagination were positively correlated with the EHQ ‘Feelings’ subscale, and each personality trait was positively correlated with the ‘Knowledge’ subscale (p<0.05), with all correlations being weak (r=0.09–0.15) None of the IPIP’s subcategories was correlated with ‘Problems’ subscale. The BDI-II scale was positively correlated with the EHQ ‘Feelings’ and ‘Problems’ subscale, but weakly (r=0.12−0.15), and negatively correlated with the ‘Knowledge’ subscale (p=0.004, p<0.0001 and p=0.001 respectively). The OCI-R total score positively correlated with the EHQ ‘Feelings’ and ‘Problems’ subscale (p=0.005 and p<0.0001 respectively) in a weak manner (r=0.02–0.17). The ‘Symptom’ subscale of the BITE questionnaire positively correlated with the EHQ ‘Feelings’ and ‘Problems’ subscale, but negatively with the ‘Knowledge’ subscale (p<0.0001), while the ‘Severity’ subscale of the BITE questionnaire positively correlated with the EHQ ‘Feelings’ subscale, but negatively with the ‘Knowledge’ subscale (p=0.001 and p<0.0001 respectively; r=−0.19–0.26). The EAT total score and subscales negatively correlated with all three EHQ subscales (p<0.05) except for the ‘Important others’ subscale with the EHQs’ ‘Feelings’ subscale and ‘Food preoccupation’ subscale with the EHQ knowledge subscale. These correlations were weak to moderate (r = −0.10 to −0.52).

## Discussion

The aim of this study was to translate and validate the Eating Habits Questionnaire in the Greek language, and investigate its psychometric properties in adults of the general population.

EFA results supported the 3-factor structure, and the 21-item version of EHQ showed satisfactory goodness-of-fit, in line with the original survey, and other validation efforts. The internal consistency, which was evaluated with Cronbach’s alpha coefficient, was found satisfying, with subscale alphas varying from 0.80 to 0.82, within the range (0.75–0.90) of other validation attempts (Mohamed Halim *et al*., 2020). The high correlation coefficient value signified the convergence of the same construct. Most EHQ items demonstrated an acceptable performance, displaying good item-total correlation coefficients, implying that all items were important components of the construct.

Criterion validity was determined by the strength of correlation between the EHQ subscales and other constructs related to orthorexia nervosa. Consistently with the original study, the subscales correlated more strongly with the questionnaires regarding eating pathology (BITE, EAT-13) than with those of obsessive-compulsive and depressive symptomatology (OCI-R, BDI-II), and personality traits (IPIP). The ‘Problems’ subscale showed no significant correlations with personality traits, very weak correlations with OCD and depressive symptomatology, and very weak to moderate correlations with eating pathology. However, the original study showed a positive correlation of the ‘Problems’ subscale with the EAT-26 scores, while this study demonstrated an up to moderate negative correlation with all the variables of the Greek version of EAT-13. This finding was supportive of other research outcomes; a study which evaluated the prevalence of ON in young adults (18–23 years of age) found a significant negative correlation between EAT-40 and ORTO-15 scores (Sanlier *et al*., 2016). The ‘Knowledge’ subscale was related with personality traits and depression, contradicting the original study. However, most of the parameters evaluating abnormal eating behavior, were in line with the original study’s findings. Very weak correlations were found between the ‘Feelings’ subscale and OCD and depression evaluation, partially contradicting the original study, which reported no correlation. However, the agreement was profound regarding the correlations with eating pathology constructs. The relationship between ON and eating pathology is presumed to be mediated by obsessive and compulsive characteristics. The fact that ON was not strongly related to OCD symptomatology contradicts existing findings which have shown that orthorexic eating and obsessive-compulsive disorder are significantly correlated, irrespectively of the severity of the disorder (Yılmaz *et al*., 2020). This discrepancy between severity of OCD symptoms and ON, highlights the fact that ON is leaning more towards eating disorders that the OCD spectrum, verifying the stronger relation of EHQ with eating behaviour instruments found in this study.

The results showed no significant sex differences, which are in line with the current literature, though some reported discrepancies seem to depend on the measuring tool used (Strahler, 2019). Like other research, the results of this study showed that BMI was not significantly correlated with neither of the three EHQ subscales. Regarding BMI and ON dimensions, several studies indicate no significant association with BMI (Ferreira and Coimbra, 2021), or sex (Oberle *et al*., 2018).

A strength of the present study was the use of a large population sample and the assessment of test-retest reliability of the questionnaire. Nevertheless, this study bears certain limitations. There may be some bias due to the self-report nature of the data, in the context of the inherent trend for socially desirable responses. This falls within all responses and mainly the weight and height measurements for the compute of body mass index. Besides, the voluntary participation in the research could lead to selection bias, as subjects were not invited to participate following the standard randomization method.

To conclude, our findings support that the Greek version of 21-item Eating Habits Questionnaire demonstrates good psychometric properties. Its internal consistency, test-retest reliability and criterion validity were sufficient. Orthorexia nervosa is an emerging health issue, and thereby, the validated Greek version of EHQ could be utilized as a useful tool for application in research and clinical practice. Future research should focus on clinical groups, specifically on individuals with eating or OCD spectrum disorders and adolescent populations which are considered to be at risk.

## Figures and Tables

**Table 1.** Participants’ sociodemographic characteristics, scales’ and subscales’ scores.

Sex	N (%)
- Female	508 (92.2)
- Male	43 (7.8)
Age Categories	N (%)
- 18–25 years	73 (13.2)
- 26–35 years	234 (42.5)
- 36–45 years	204 (37.0)
- 46–65 years	40 (7.3)
Marital status	N (%)
- Single	266 (48.3)
- Married	245 (44.5)
- Divorced	31 (5.6)
- Widowed	9 (1.6)
Educational level	N (%)
- High school	65 (11.8)
diploma	88 (16.0)
- IEK-TEE degree	258 (46.8)
- TEI-AEI degree	140 (25.4)
- Master’s degree	
Ethnicity	N (%)
- Greek	545 (99.3)
- Other	4 (0.7)
Weight satisfaction	N (%)
- Not at all	176 (31.9)
- Relatively	274 (49.7)
- absolutely	101 (18.3)
ED History	N (%)
- Yes	94 (17.1)
- No	457 (82.9)
BMI	22.73 (5.79)
	24.04 (4.79)
IPIP score	IQR/SD
- Extraversion	33.00 (8.00)
	32.77 (6.15)
- Agreeableness	36.00 (4.00)
	36.19 (3.07)
- Conscientiousness	35.00 (10.00)
	34.75 (6.27)
- Emotional stability	28.00 (8.00)
	28.08 (5.57)
- Intellect/ Imagination	35.00 (5.00)
	35.29 (3.56)
BDI score	10.00 (11.00)
	11.63 (8.30)
BITE score	14.00 (12.00)
	15.38 (7.70)
- Severity	3.00 (3.00)
	3.71 (1.58)
OCI-R score	41.00 (18.00)
	41.27 (12.08)
- Washing	5.00 (4.00)
	5.95 (2.94)
- Checking	6.00 (4.00)
	7.03 (3.01)
- Ordering	8.00 (5.00)
	8.26 (3.18)
- Obsessing	7.00 (5.00)
	7.54 (3.28)
- Hoarding	7.00 (5.00)
	7.54 (3.11)
- Neutralizing	4.00 (3.00)
	4.95 (2.32)
EAT score	55.00 (11.00)
	55.00 (8.73)
- Dieting	16.00 (5.00)
	16.12 (3.77)
- Food preoccupation	24.00 (10.00)
	22.97 (7.08)
- Important others	17.00 (3.00)
	15.91 (2.78)
EHQ score	
- Feelings	11.00 (5.00)
	10.77 (3.06)
- Knowledge	10.00 (5.00)
	10.60 (3.23)
- Problems	15.00 (5.00)
	16.25 (4.71)

IQR: Interquartile Range; SD: Standard Deviation; ED: Eating Disorder; BMI: Body Mass Index; IPIP: International Personality Item Pool; BDI: Beck’s Depression Inventory; BITE: Bulimic Investigatory Test, Edinburg; OCI-R: Obsessive Compulsive Disorder – Revised, EAT: Eating Attitudes Test; EHQ: Eating Habits Questionnaire

**Table 2.** Rotated factor loadings of the principal components analysis (PCA) for 21 items (N= 551)

Items	EHQ Knowledge	EHQ Problems	EHQ Feelings
1. I am more informed than others about healthy eating	0.52		
3. The way my food is prepared is important in my diet	0.721		
5. My eating habits are superior to others	0.761		
11. My diet is better than other people’s diets	0.736		
21. I prepare food in the most healthful way	0.667		
2. I turn down social offers that involve eating unhealthy food.		0.348	
4. I follow a diet with many rules		0.387	
6. I am distracted by thoughts of eating healthily		0.574	
7. I only eat what my diet allows		0.351	
8. My healthy eating is a significant source of stress in my relationships		0.693	
10.My diet affects the type of employment I would take		0.554	
13. In the past year, friends or family members have told me that I’m overly concerned with eating healthily.		0.559	
14. I have difficulty finding restaurants that serve the foods I eat		0.597	
16. Few foods are healthy for me to eat		0.618	
17. I go out less since I began eating healthily		0.582	
18. I spend more than three hours a day thinking about healthy food.		0.685	
20. I follow a health-food diet rigidly		0.415	
9. I have made efforts to eat more healthily over time			0.694
12. I feel in control when I eat healthily			0.817
15. Eating the way I do gives me a sense of satisfaction			0.842
19. I feel great when I eat healthily			0.814
Eigenvalues	6.269	2.121	1.731
% of Variance	29.854	10.099	8.244
Croncbach’s alpha	0.796	0.82	0.817

EHQ: Eating Habits Questionnaire

**Table 3.** Correlations (Spearman’s rho) between EHQ subscales.

	EHQ Feelings	EHQ Knowledge	EHQ Problems
EHQ Feelings	1	0.443**	0.477**
EHQ Knowledge		1	0.521**
EHQ Problems			1

**Table 4.** Descriptive characteristics of the three subscales of EHQ (1st and 2nd measurement) and test-retest reliability.

Subscale	Items	Range	Mean	SD	Minimum	Maximum	p-value
EHQ Feelings 1st time	4	1–16	10.77	3.06	4	16	0.388
EHQ Feelings 2nd time			11.2	3.49	4	16	
EHQ Knowledge 1st time	5	1–20	10.6	3.23	5	20	0.596
EHQ Knowledge 2nd time			12	3.88	5	20	
EHQ Problems 1st time	12	1–48	16.25	4.71	12	39	0.666
EHQ Problems 2nd time			15.5	4.38	12	31	

SD: standard deviation, Wilcoxon, p<0.05; EHQ: Eating Habits Questionnaire

**Table 5.** Associations between EHQ subscales and other study measurements.

Study measurements	Categories	EHQ Feelings	EHQ Knowledge	EHQ Problems
Sex Median (IQR) Mean (SD)	Males	11.00 (4.00)	11.00 (5.00)	15.00 (7.00)
		10.09 (3.27)	10.35 (2.95)	16.37 (4.85)
	Females	11.00 (5.00)	10.00 (5.00)	15.00 (5.00)
		10.83 (3.04)	10.62 (3.25)	16.23 (4.70)
	p-value	0.194	0.597	0.898
Education level Median (IQR) Mean (SD)	High School	11.00 (5.50)	11.00 (7.00)	23.00 (13.00)
		10.00 (3.53)	9.40 (3.64)	19.80 (6.76)
	Lyceum	9.00 (5.00)	10.00 (4.00)	14.00 (3.00)
		9.80 (3.25)	10.02 (3.10)	15.35 (3.82)
	Vocational Training	11.50 (5.00)	9.50 (4.00)*	15.00 (4.75)
		10.82 (3.15)	9.77 (3.00)	15.78 (4.34)
	Tertiary Education	11.00 (4.00)	11.00 (5.00)	15.00 (5.00)
		10.88 (2.89)	10.79 (3.16)	16.45 (4.80)
	MSc/PhD	11.00 (4.75)	11.00 (4.00)*	15.00 (5.00)
		10.99 (3.16)	11.06 (3.42)	16.42 (4.98)
	p-value	0.116	0.016	0.254
Marital status Median (IQR) Mean (SD)	Single	11.00 (4.25)	10.00 (4.00)	15.00 (4.00)
		10.96 (3.00)	10.36 (3.18)	16.09 (4.43)
	Married	11.00 (5.00)	10.00 (4.50)	15.00 (4.00)*
		10.46 (3.03)	10.79 (3.21)	16.13 (4.93)
	Divorced	12.00 (5.00)	11.00 (6.00)	17.00 (7.00)
		11.06 (3.64)	10.94 (3.41)	17.32 (4.60)
	Widowed	13.00 (5.50)	11.00 (7.50)	18.00 10.00)*
		12.56 (2.83)	11.44 (4.12)	20.22 (5.31)
	p-value	0.075	0.351	0.015
IPIP Spearman rho p-value	Extraversion	0.062	0.086*	0.019
		0.145	0.043	0.664
	Agreeableness	0.108*	0.093*	0.059
		0.012	0.03	0.168
	Conscientiousness	0.069	0.181**	0.081
		0.107	0	0.057
	Emotional stability	−0.05	0.093*	−0.009
		0.241	0.029	0.828
	Intellect/ Imagination	0.095*	0.150*	0.051
		0.025	0	0.236
BDI-II Spearman rho p-value	Total	0.122**	−0.135**	0.149**
		0.004	0.001	0
OCI-R Spearman rho p-value	Total	0.121**	−0.057	0.156**
		0.005	0.185	0
	Washing	0.019*	0.032	0.165**
		0.01	0.459	0
	Checking	0.051	−0.04	0.08
		0.228	0.347	0.059
	Ordering	0.068	−0.013	0.091*
		0.11	0.766	0.033
	Obsessing	0.111**	−0.119**	0.108*
		0.009	0.005	0.011
	Hoarding	0.072	−0.046	0.083
		0.09	0.279	0.051
	Neutralizing	0.023	−0.046	0.101*
		0.591	0.28	0.018
BITE Spearman rho p-value	Symptom	0.262**	−0.193**	0.175**
		0	0	0
	Severity	0.148**	−0.204**	0.064
		0.001	0	0.132
EAT Spearman rho p-value	Total	−0.512**	−0.283**	−0.514**
		0	0	0
	Dieting	−0.354**	−0.524**	−0.442**
		0	0	0
	Food preoccupation	−0.448**	−0.063	−0.365**
		0	0.141	0
	Important others	0.039	−0.116**	−0.100*
		0.365	0.007	0.019
BMI Spearman rho p-value		0.132	−0.319	0.112
		0.486	0.085	0.557

Mann-Whitney, Kruskal Wallis, One way ANOVA, Spearman’s rho, p<0.05; IPIP: International Personality Item Pool; BDI: Beck’s Depression Inventory; BITE: Bulimic Investigatory Test, Edinburg; OCI-R: Obsessive Compulsive Disorder – Revised, EAT: Eating Attitudes Test; EHQ: Eating Habits Questionnaire; BMI: Body Mass Index

## Key Points

- When healthy eating becomes obsessive, psychological disturbances may emerge
- The overwhelming preoccupation with health eating may lead to orthorexia nervosa
- The Eating Habits Questionnaire assesses orthorexic characteristics
- The Greek version of the Eating Habits Questionnaire demonstrated good psychometric properties in adults.

## References

[R1] American Psychiatric Association (2013) Diagnostic and Statistical Manual of Mental Disorders American Psychiatric Association, Fifth Edition.

[R2] Barnes MA, Caltabiano ML (2017) The interrelationship between orthorexia nervosa, perfectionism, body image and attachment style. Eat Weight Disord 22 (1), 177–184. 10.1007/s40519-016-0280-x 27068175

[R3] Beck AT, Steer RA, Carbin MG (1988) Psychometric properties of the Beck Depression Inventory: Twenty-five years of evaluation. Clinical Psychology Review 8 (1), 77–100. 10.1016/0272-7358(88)90050-5

[R4] Brytek-Matera A, Staniszewska A, Hallit S (2020) Identifying the Profile of Orthorexic Behavior and “Normal” Eating Behavior with Cluster Analysis: A Cross-Sectional Study among Polish Adults. Nutrients 12 (11), 3490. 10.3390/nu12113490 33202994 PMC7696927

[R5] Cena H, Barthels F, Cuzzolaro M, Bratman S, Brytek-Matera A, (2019) Definition and diagnostic criteria for orthorexia nervosa: a narrative review of the literature. Eat Weight Disord 24 (2), 209–246. 10.1007/s40519-018-0606-y 30414078

[R6] Costello AB, Osborne J (2005) Best practices in exploratory factor analysis: four recommendations for getting the most from your analysis. Practical Assessment 10.7275/JYJ1-4868

[R7] Douka A, Grammatopoulou E, Skordilis E, Koutsouki D (2009) Factor analysis and cut-off score of the 26-item eating attitudes test in a Greek sample. jbe 5 (1) 10.4127/jbe.2009.0025

[R8] Douma ER, Valente M, Syurina EV (2021) Developmental pathway of orthorexia nervosa: Factors contributing to progression from healthy eating to excessive preoccupation with healthy eating. Experiences of Dutch health professionals. Appetite 158, 105008. 10.1016/j.appet.2020.105008 33069774

[R9] Ferreira C, Coimbra M (2021) To further understand orthorexia nervosa: DOS validity for the Portuguese population and its relationship with psychological indicators, sex, BMI and dietary pattern. Eat Weight Disord 26 (7), 2127–2134. 10.1007/s40519-020-01058-4 33140378

[R10] Foa EB, Huppert JD, Leiberg S, Langner R, Kichic R, (2002) The Obsessive-Compulsive Inventory: development and validation of a short version. Psychol Assess 14 (4), 485–496. 12501574

[R11] Garner DM, Olmsted MP, Bohr Y, Garfinkel PE (1982) The Eating Attitudes Test: psychometric features and clinical correlates. Psychol. Med 12 (4), 871–878. 10.1017/S0033291700049163 6961471

[R12] Giannakou M, Roussi P, Kosmides M-E, Kiosseoglou G, Adapomopoulou A, (2013) Adaptation of the Beck Depression Inventory-II to Greek population. Hellenic Journal of Psychology 10 (2), 120–146.

[R13] Gleaves DH, Graham EC, Ambwani S (2013) Measuring “orthorexia”: Development of the Eating Habits Questionnaire. The International Journal of Educational and Psychological Assessment 12 (2), 1–18.

[R14] Hayes O, Wu MS, De Nadai AS, Storch EA (2017) Orthorexia Nervosa: An Examination of the Prevalence, Correlates, and Associated Impairment in a University Sample. J Cogn Psychother 31 (2), 124–135. 10.1891/0889-8391.31.2.124 32755933

[R15] Henderson M, Freeman CPL (1987) A Self-rating Scale for Bulimia the ‘BITE’. Br J Psychiatry 150 (1), 18–24. 10.1192/bjp.150.1.18 3651670

[R16] Kiss-Leizer M, Rigó A (2019) People behind unhealthy obsession to healthy food: the personality profile of tendency to orthorexia nervosa. Eat Weight Disord 24 (1), 29–35. 10.1007/s40519-018-0527-9 29934757

[R17] Kokka I, Mourikis I, Michou M, Vlachakis D, Darviri C, (2021) Validation of the Greek Version of Social Media Disorder Scale. Adv Exp Med Biol, Vol, 1338, 107–116. 10.1007/978-3-030-78775-2_13 34973015

[R18] McComb SE, Mills JS (2019) Orthorexia nervosa: A review of psychosocial risk factors. Appetite 140, 50–75. 10.1016/j.appet.2019.05.005 31075324

[R19] Mohamed Halim Z, Dickinson KM, Kemps E, Prichard I (2020) Orthorexia nervosa: examining the Eating Habits Questionnaire’s reliability and validity, and its links to dietary adequacy among adult women. Public Health Nutr. 23 (10), 1684–1692. 10.1017/S1368980019004282 32204747 PMC10200377

[R20] Oberle CD, Samaghabadi RO, Hughes EM (2017) Orthorexia nervosa: Assessment and correlates with gender, BMI, and personality. Appetite 108, 303–310. 10.1016/j.appet.2016.10.021 27756637

[R21] Oberle CD, Watkins RS, Burkot AJ (2018) Orthorexic eating behaviors related to exercise addiction and internal motivations in a sample of university students. Eat Weight Disord 23 (1), 67–74. 10.1007/s40519-017-0470-1 29260414

[R22] Rudolph S (2018) The connection between exercise addiction and orthorexia nervosa in German fitness sports. Eat Weight Disord 23 (5), 581–586. 10.1007/s40519-017-0437-2 28884261

[R23] Sanlier N, Yassibas E, Bilici S, Sahin G, Celik B (2016) Does the rise in eating disorders lead to increasing risk of orthorexia nervosa? Correlations with gender, education, and body mass index. Ecology of Food and Nutrition 55 (3), 266–278. 10.1080/03670244.2016.1150276 26979290

[R24] Simos G, Zikopoulou O, Nisyraiou A, Zafiropoulos K (2019) Psychometric Properties of the Greek Version of the Obsessive-Compulsive Inventory-Revised in a Non-Clinical Young Adult Sample. PSYCH 10 (16), 2247–2265. 10.4236/psych.2019.1016142

[R25] Strahler J (2019) Sex differences in orthorexic eating behaviors: A systematic review and meta-analytical integration. Nutrition 67–68, 110534. 10.1016/j.nut.2019.06.015 31525607

[R26] Yılmaz H, Karakuş G, Tamam L, Demirkol ME, Namlı Z, (2020) Association of Orthorexic Tendencies with Obsessive-Compulsive Symptoms, Eating Attitudes and Exercise. NDT 16, 3035–3044. 10.2147/NDT.S280047 PMC7751779 33364760

[R27] Ypofanti M, Zisi V, Zourbanos N, Mouchtouri B, Tzanne P, (2015) Psychometric properties of the International Personality Item Pool Big-Five personality questionnaire for the Greek population. Health Psych Res 3 (2) 10.4081/hpr.2015.2206 PMC4768534 26973962

